# Cannabidiol Extracted from *Cannabis sativa* L. Plant Shows Neuroprotective Impacts Against 6-HODA-Induced Neurotoxicity via *Nrf2* Signal Transduction Pathway

**DOI:** 10.5812/ijpr-160499

**Published:** 2025-05-26

**Authors:** Afsaneh Esfandi, Ali Mehrafarin, Sepideh Kalateh Jari, Hassanali Naghdi Badi, Kambiz Larijani

**Affiliations:** 1 Department of Horticultural Science and Agronomy, Science and Research Branch, Islamic Azad University, Tehran, Iran; 2 Medicinal Plants Research Center, Shahed University, Tehran, Iran; 3 Department of Agronomy and Plant Breeding, Faculty of Agriculture, Shahed University, Tehran, Iran; 4 Department of Chemistry, Science and Research Branch, Islamic Azad University, Tehran, Iran

**Keywords:** Cannabidiol, Cell, Gene, Anti-oxidant Capacity

## Abstract

**Background:**

As a prevalent neurodegenerative illness, Parkinson's disease (PD) is associated with serious disability and reduced quality of patients' lives. Therefore, finding new adjuvant treatment approaches that can improve patients' quality of life is crucial.

**Objectives:**

This study evaluated the impacts of cannabidiol (CBD) on the PC12 cell line and elucidated its mechanism of action, emphasizing the antioxidant pathway.

**Methods:**

First, CBD was extracted from the hemp plant. Then, the cells were treated with CBD at different dosages. After treatment, the cells were exposed to 6-HODA, and cell viability and apoptosis, reactive oxygen species (ROS) content, total antioxidant capacity, lipid peroxidation, super oxide dismutase (SOD) and GSH levels, as well as the *Nrf2*, *Bax*, *Bcl-2*, and *Casp3* genes' expressions were measured.

**Results:**

Cannabidiol augmented the cell viability and decreased the apoptosis rates of 6-HODA-exposed PC12 cells. Also, pretreatment of PC12 cells with CBD was associated with decreases in ROS and malondialdehyde (MDA) contents, and an improvement in total antioxidant capacity and SOD and GSH activities were also seen. In addition, CBD overexpressed *Nrf2* and *Bcl-2* genes in 6-HODA-exposed PC12 cells and, on the other hand, prevented the upregulation of *Bax* and *Casp3*.

**Conclusions:**

Overall, it was concluded that CBD has neuroprotective impacts against 6-HODA-induced neurotoxicity via the *Nrf2* signal transduction pathway.

## 1. Background

An increasing trend of Parkinson's disease (PD), as the neurodegenerative illness, has been reported, and it is more prevalent in countries with high human development and sociodemographic indicators (1). The degeneration of dopaminergic neurons is the main pathophysiological attribute of PD, which is accompanied by a decrease in dopamine secretion (2), and over time, motor (tremor, muscle rigidity, slowness of movement, and imbalance) and non-motor (postural hypotension, depression, sleep disorders, hallucinations, fatigue, and pain) symptoms appear (3). The pathophysiology of this disease is not well known. However, mitochondrial complex I damage results from oxidative stress and overproduction of reactive oxygen species (ROS) involve in developing this disease (4). In addition, inflammation, dysfunction of mitochondria, and proteasomal defects play a key role in the initiation of substantia nigra death, leading to the death of dopaminergic neurons and the Lewy bodies (LB) formation (5, 6). These bodies contain α-synuclein, the accumulation of which is associated with increased dopaminergic neuron degeneration (7), neuroinflammation, and disease progression (8). Elevated secretion of the cytokines TNF-α, IL-6, and IL-1β is associated with elevated neuronal inflammation, which is accompanied by decreased expression of anti-inflammatory mediators including IL-10 (9). Thus, both inflammation and oxidative stress are implicated in PD pathogenesis. Levodopa, as a dopamine precursor, has been the frontline treatment for this disease (3), and although it improves symptoms in the short term, it requires high doses in the long term, which is associated with side effects resulting in reduced patients' life quality (10). Therefore, finding new therapeutic approaches for PD is of great importance. Recently, targeting the *Nrf2* pathway, which is involved in antioxidant and inflammatory pathways, has been considered a novel therapeutic option for PD (11). This pathway plays a pivotal role in maintaining mitochondrial function and ROS scavenging, and thus, prevents dopaminergic neuron degeneration (12). Therefore, drugs targeting and activating the *Nrf2* pathway have great potential in the treatment of PD. Recently, natural products have gained prominence in neurodegenerative disease research, including PD, and have demonstrated neuroprotective effects — primarily through their anti-inflammatory and antioxidant properties mediated by activation of the *Nrf2* pathway (13). Cannabidiol (CBD) is a major constituent of the hemp plant (*Cannabis sativa*) (14), and its PD-symptom alleviating effects have recently attracted the attention of researchers. For example, CBD showed neuroprotective impacts against neurotoxicity induced by MPTP in vivo (15). In addition, the anti-inflammatory impacts of this compound have been demonstrated by reducing NF-κB and COX-2 levels through affecting PPARγ receptors (16). Also, CBD reduced oxidative stress levels and prevented the decrease in dopamine levels (17). Furthermore, CBD prevented microglia-induced neuroinflammation and prevented oxidative stress (18). Both anti-inflammatory and antioxidant effects of CBD appear to play a pivotal role in inducing neuroprotective effects. Furthermore, it has been shown that CBD can act as a regulator of CB1 and CB2 receptors in the central nervous system, exhibiting neuroprotective effects (19) and is recommended in the treatment of diseases such as multiple sclerosis, epilepsy, and PD (20). Interestingly, CBD was recently shown to be able to reduce the aggregation of alpha-synuclein (an important factor in the pathogenesis of PD) in a *C. elegans* model, and its administration was associated with a reduction in lipid peroxidation and an upregulation of *SOD-3* expression (21). Cannabidiol also demonstrated neuroprotective effects through anti-inflammatory and antioxidant properties in an animal model of Alzheimer’s disease, which was associated with improved cognitive function in the animals (22). Importantly, CBD has far fewer side effects compared to chemical drugs and has been shown to be well tolerated (23). These findings indicate the potent antioxidant properties of this compound. Notably, activation of the *Nrf2* pathway plays a key role in antioxidant defense, and it is unknown whether CBD can exert antioxidant effects by activating this pathway.

## 2. Objectives

This research was conducted for the first time to evaluate the impacts of CBD on the 6-OHDA-induced neurotoxicity in the PC12 cell model with an emphasis on antioxidant mechanisms exerted via the *Nrf2* pathway.

## 3. Methods

### 3.1. Plant Preparation and Cannabidiol Extraction

The detail of hemp plant preparation and CBD extraction was reported by our research group recently (14). Briefly, the hemp seeds were cultured in the greenhouse of the Institute of Medicinal Plants Research in Karaj-Iran, and the mature plants were harvested in the middle of September. The microwave (400W) drying method, which resulted in the highest CBD extraction (431.3 ± 1.25 µg/g) (14), was used in the current study. After drying and in order to prepare the ethanolic extract, 20 g of cannabis plant was completely powdered and dissolved in 96% ethanol (200 mL) under shaking conditions for 4 h. Then, the solution was filtered using Whatman paper No. 1 and a 0.2 micron filter. The ethanol solution was evaporated at 40°C, and the dry extract was dissolved in a water/methanol ratio of 1:5. Then, it was transferred to the UHPLC device (24). UHPLC/UV device was used for extraction using HALO column (C18 Fused-Core), with a HALO guard column. Three solvents of acetic acid dissolved in water (A, 0.1%), acetic acid dissolved in acetonitrile (B, 0.1%), and methanol with 0.25 mL/min rate were used. The temperature of the column was set at 30°C. UV-Vis scan mode was used for data acquisition. The extracted CBD was dissolved in ethanol/saline solvent in a 1:18 ratio (25).

### 3.2. Cell Preparation, Culture, and Treatment

PC12 cells were obtained from the Pasteur Institute of Iran and immediately seeded in DMEM (Gibco, USA). The medium was supplemented with two antibiotics of penicillin (100 U/mL) and streptomycin (100 μg/mL) for preventing contamination and 10% FBS for providing essential factors for cell growth. The plates were placed at 37°C and 95% CO_2_. Cells were grouped as follows: (1) Control group treated with ethanol/saline solvent (1:18 ratio) (Ctl); (2) cells treated with 6-hydroxydopamine (6-OHDA, 100 µM); (3) cells pre-treated with 1 µM CBD and treated with 6-OHDA; (4) cells pre-treated with 5 µM CBD and treated with 6-OHDA; (5) cells pre-treated with 10 µM CBD and treated with 6-OHDA. It is worth noting that the selection of CBD doses was based on prior studies. For example, Gugliandolo et al. demonstrated that a dose of 10 μM CBD improved the viability of SH-SY5Y cells treated with MPP^+^ (26), whereas no significant effect on cell viability was observed at concentrations of 1 and 2.5 μM CBD (27). However, 1 μM CBD showed protective effects against 100 μM MPP^+^ (28). Considering that CBD exhibits neurotoxic effects at higher concentrations, a dose range of 1 to 10 μM was selected for this study.

### 3.3. Viability

The viability percentages of PC12 cells were evaluated by the MTT assay. For this, initially 10^5^ cells were seeded in each well of the plate and placed at 37°C overnight. In the next step, the cells were exposed to the above-mentioned concentrations of CBD (Section 2.2) and 100 μM 6-OHDA for 24 h. Then, 10 μL of MTT solution (Sigma, USA) was added to each well and incubated for 3 hours. Subsequently, the culture medium was removed, and 100 μL of DMSO was added to each well. Finally, the optical density was measured at 490 nm (29).

### 3.4. Reactive Oxygen Species

For this purpose, we used the DCF-DA method. Briefly, the cells were seeded in plates for 24 h, then, they were treated with CBD and 6-OHDA (100 μM). After emptying the culture medium and washing the cells with PBS, they were incubated with DCF-DA for half an hour. Finally, the fluorescence level was read by ELISA reader at excitation 485/20 and emission 528/20 (30).

### 3.5. Apoptosis

The apoptosis and necrosis rates of cells were measured by flow cytometry and V-FITC/PI staining method. In short, after incubation with CBD and 6-OHDA, the cells were exposed to PI and V-FITC (5 μL) for 900 s at 37°C. Then, cell apoptosis was measured using a flow cytometry device (30).

### 3.6. Lipid Peroxidation and Antioxidant Capacity

Cells were cultured in 25 cm^2^ flasks and after overnight treatment with CBD, they were treated with 6-OHDA. In the next step, cells were centrifuged at 1200 g for 6 min, and malondialdehyde (MDA) content and antioxidant capacity of PC12 cells were measured as follows. Malondialdehyde was evaluated as an indicator of lipid peroxidation. For this purpose, the formation of a bond between MDA and thiobarbituric acid reactive substances (TBARS) under acidic conditions and high temperature, and reading OD at a wavelength of 532 nm by spectrophotometer were used (31). Total antioxidant power was measured using the FRAP method (32). The basis of this method is the ability to reduce ferric ions (Fe^3+^) and convert them to ferrous ions (Fe^2+^) under acidic conditions and in the presence of tripyridyl triazine (TPTZ). In this reaction, a blue complex was formed, and the OD (593 nm) was measured.

### 3.7. Superoxide Dismutase and Reduced Glutathione

We used kits for measuring the contents of GSH and super oxide dismutase (SOD) in PC12 cells based on the manufacturer's instructions (Sigma, USA). Briefly, the PC12 cells (10^5^ cell/well) were seeded in the plate and pre-treated with CBD and followed by exposure to 6-OHDA overnight. Then, after preparing the homogenate, the levels of activities of SOD and GSH were evaluated by the aforementioned kits using a spectrophotometer and reading OD at 450 nm.

### 3.8. Gene Expression

#### 3.8.1. RNA Extraction and cDNA Synthesis

Commercial ParsTous kit (Iran) was used for extracting RNA, and all steps were done based on the manufacturer's instructions. Then, NanoDrop (OD ratio 260/280) and agarose gel electrophoresis (observation of 18s and 28s bands) were used for evaluating the quantity and quality of extracted RNA, respectively. After ensuring optimal RNA quantity and quality, cDNA synthesis was performed using a commercial kit (ParsTous, Iran). The synthesized cDNA was stored at -80°C for use in subsequent stages of the experiment.

#### 3.8.2. Primer Design

The primers for the studied genes (*Nrf2*, *Bax*, *Bcl-2*, and *Casp3*) were designed using Primer 3 software. Then, to ensure the accuracy, the designed sequences were blasted on the NCBI website. After ensuring the accuracy of the gene sequences, the primers were synthesized (Table 1). 

**Table 1.** The Primers' Sequences of *Nrf2*, *Bax*, *Bcl-2* and *Casp3* Genes

Genes	Sequences (5'-3')
***Nrf2***	
Forward	CGGTATGCAACAGGACATTG
Reverse	GTTTGGCTTCTGGACTTGGA
***B** **ax***	
Forward	GTGGATGACTGAGTACCTGAAC
Reverse	GCCAGGAGAAATCAAACAGAGG
***B** **cl-2***	
Forward	GAGCAGATCATGAAGACAGGG
Reverse	ATGCGCTTGAGACACTCG
***Casp3***	
Forward	GGAAGCGAATCAATGGACTCTGG
Reverse	GCATCGACATCTGTACCAGACC
***GAPDH***	
Forward	GTGAACCATGAGAAGTATGACAAC
Reverse	CATGAGTCCTTCCACGATACC

#### 3.8.3. RT-PCR

To measure the mRNA levels of the studied genes, RT-PCR and master mix SYBR Green (ParsTous, Iran) were used according to the manufacturer's instructions. The temperature-time program was one cycle of 95°C for 600 s, 45 cycles of 95°C for 30 s, 60°C for 25 s, and 72°C for 35 s, and a last cycle of 72°C for 600 s. The resulting data were analyzed using the 2^-ΔΔCT ^method.

### 3.9. Statistical Analysis

GraphPad Prism V.8 software was used for analyzing the data. The normal distribution of data was evaluated by the Kolmogorov-Smirnov test. After ensuring normal distribution, the data were analyzed using a parametric procedure, namely ANOVA, for variance analysis and followed by Tukey's post hoc test for means comparisons by considering P < 0.05 as the level of significance.

## 4. Results

### 4.1. Cell Viability and Apoptosis

**Figure 1.**
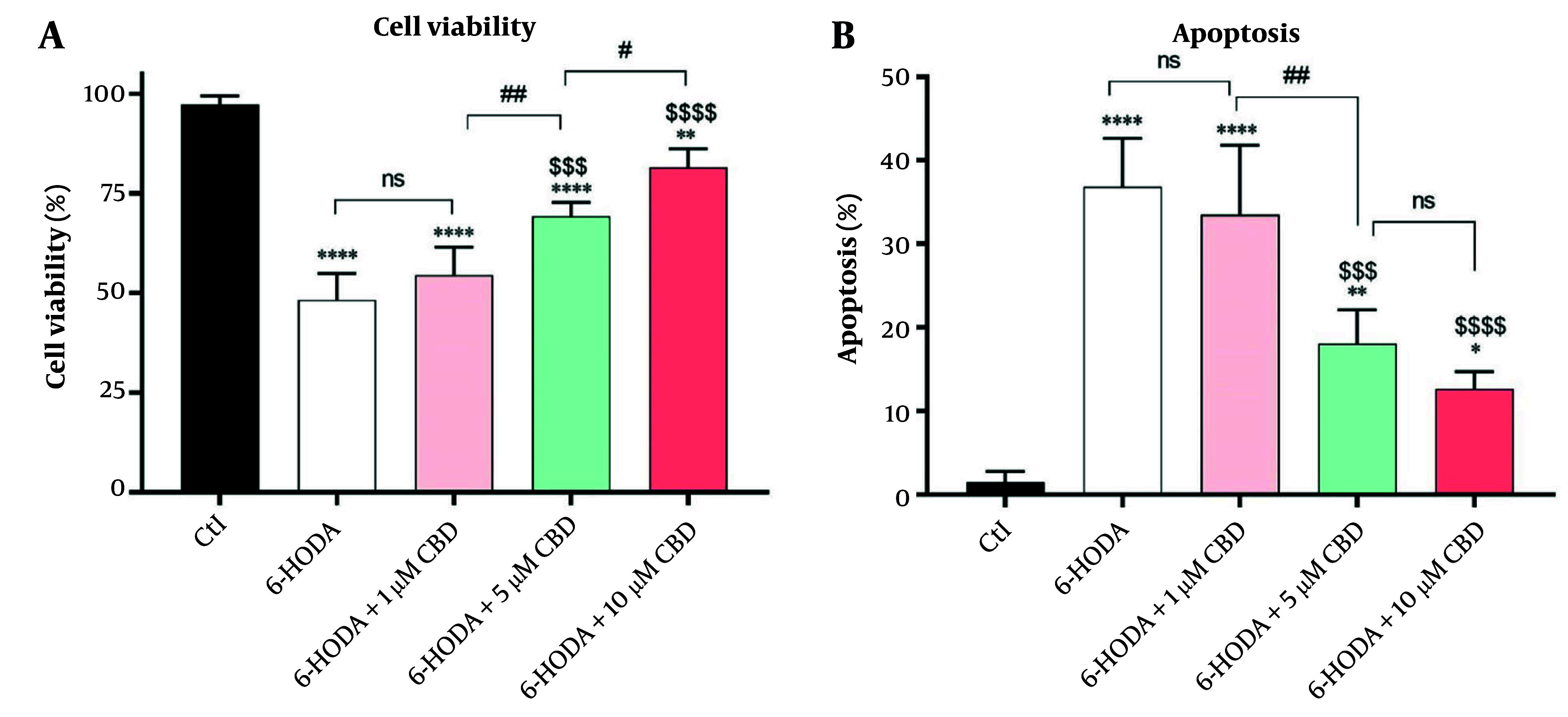
The effects of pretreatment of the PC12 cell line with cannabidiol (CBD) on the cells' viability (A) and apoptosis (B) percentages (n = 4). The cells were pre-treated with CBD for 24 h and then exposed to 6-OHDA for 24 h. **** P < 0.0001, ** P < 0.01, and * P < 0.05, compared with control cells. $$$$ P < 0.0001 and $$$ P < 0.001 compared with 6-OHDA-exposed cells. #, and ## indicate significant differences at probability levels of P < 0.05, and P < 0.01, respectively, while ns represents non-significant results.

The variance analysis showed significant differences in cell viability [F (4, 15) = 60.14, P < 0.0001] and apoptosis [F (4, 15) = 34.23; P < 0.0001] percentages among cells. A significant decrease in the PC12 cells' viability was seen after they were exposed to 6-OHDA (48.25 ± 6.65%) compared to control cells (97.25 ± 2.21%) (P < 0.0001), indicating 6-OHDA-induced neurotoxicity. However, pretreatment of PC12 cells with 5 (69.25 ± 3.50%, P < 0.001) and 10 μM (81.65 ± 4.50%, P < 0.0001) CBD for 24 h significantly prevented 6-OHDA-induced toxicity compared to the positive control. Also, there was a significant difference in the viability of PC12 cells exposed to 6-OHDA and pretreated with 5 μM and 10 μM CBD (P = 0.029), and the cell viability rate was higher in cells treated with the highest concentration of CBD (Figure 1A). Importantly, these neurotoxic effects of 6-OHDA were found to be due to the induction of apoptosis in the cells, and the highest percentage of apoptosis was observed in PC12 cells exposed to 6-OHDA (36.82 ± 5.81%), which was significantly different compared to control cells (1.5 ± 1.29%, P < 0.0001). However, CBD at concentrations of 5 and 10 μM (18.04 ± 4.05 and 12.60 ± 2.07%, respectively) was able to significantly inhibit the 6-OHDA-induced apoptosis (Figure 1B). 

### 4.2. Malondialdehyde and Reactive Oxygen Species

**Figure 2.**
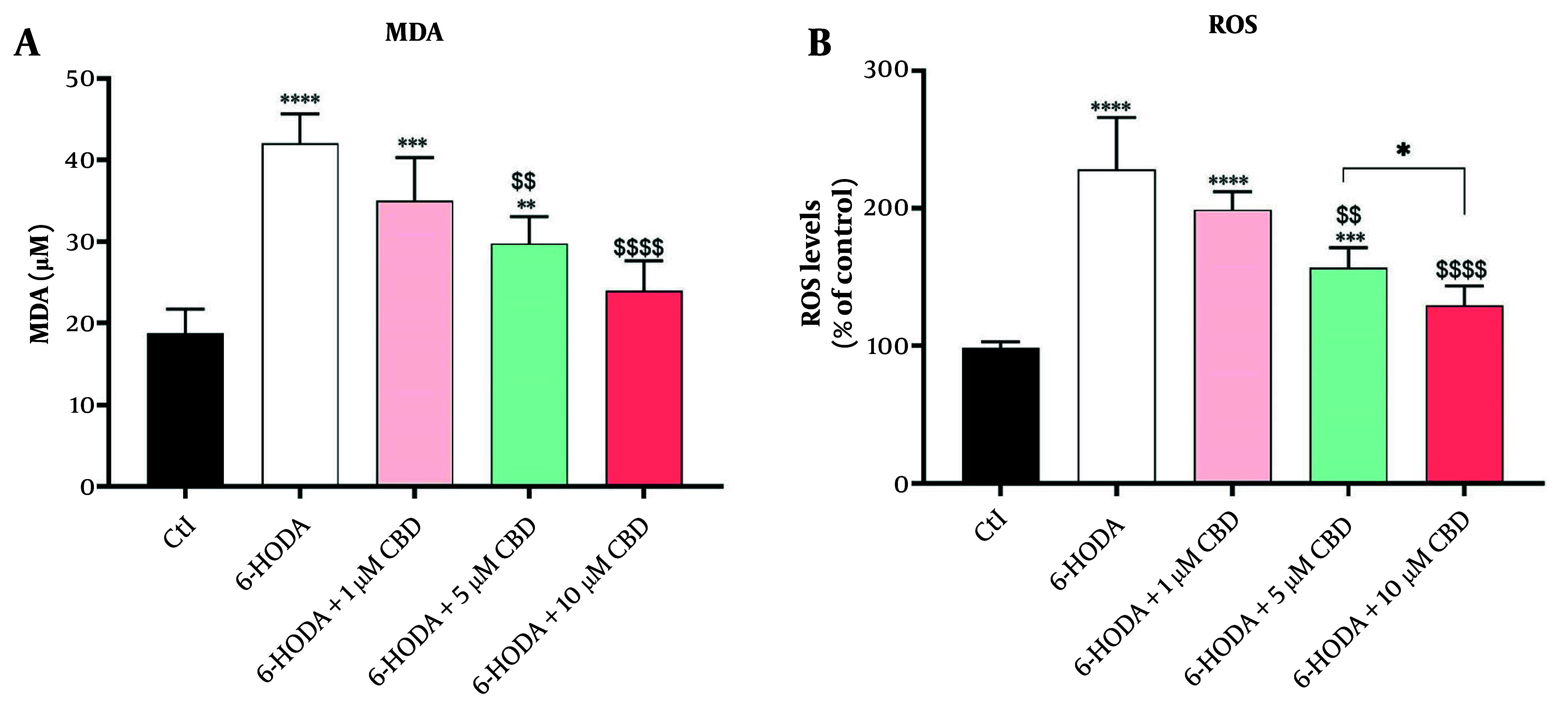
The impacts of pretreatment of PC12 cell line with cannabidiol (CBD) on the malondialdehyde (MDA) (A) and reactive oxygen species (ROS) (B) contents (n = 4). The cell pre-treated with CBD and exposed with 6-OHDA for 24 h. * Shows significant difference at the probability level of P < 0.05; **** P < 0.0001, ***P < 0.001 and **P < 0.01, compared with control cells. $$$$ P < 0.0001 and $$ P < 0.01 compared with 6-OHDA-exposed cells.

There were significant differences in MDA [F (4, 15) = 22.25; P < 0.0001] and ROS [F (4, 15) = 26.40; P < 0.0001] contents among cells. PC12 cells exposed to 6-OHDA showed significant overproductions of both MDA (42.00 ± 3.65 μM, Figure 2A) and ROS (228 ± 0.38% of control, Figure 2B) contents compared to control, indicating the induction of oxidative stress and neuronal lipid peroxidation by 6-OHDA. Although exposure of cells to 6-OHDA and 1 μM CBD did not result in a significant difference in MDA content compared to 6-OHDA alone (P = 0.128), concentrations of 5 (P = 0.003) and 10 μM (P < 0.0001) CBD significantly reduced MDA content in cells compared to cells treated with 6-OHDA (Figure 2A). This finding suggests that CBD has the ability to inhibit neuronal lipid peroxidation induced by 6-OHDA. Similarly, lower ROS content was observed in cells treated with 5 μM (P = 0.014) and 10 μM (P < 0.0001) CBD compared to PC12 cells exposed to 6-OHDA. Interestingly, there were also significant differences in MDA content in PC12 cells treated with 5 and 10 μM CBD (P = 0.039), and cells treated with higher CBD concentrations had lower ROS content compared to PC12 cells treated with 5 μM CBD (Figure 2B). 

### 4.3. Total Antioxidant Capacity, Super Oxide Dismutase, and GSH

**Figure 3.**
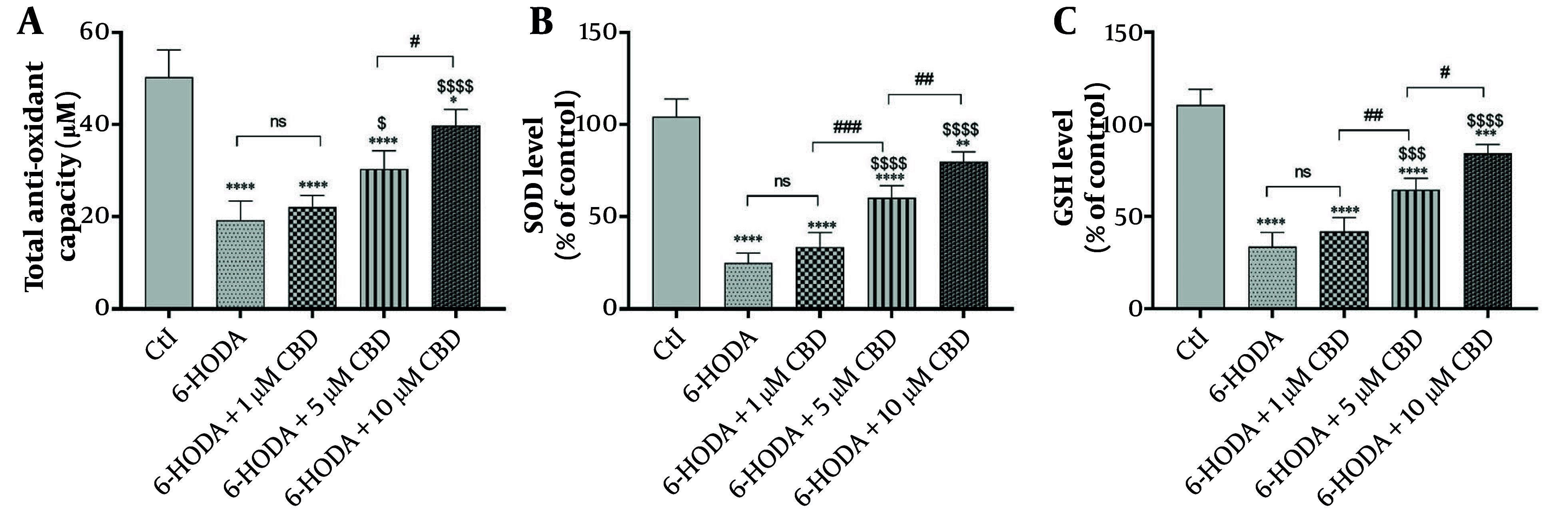
The effects of pretreatment of PC12 cell line with different concentrations of cannabidiol (CBD) on the total anti-oxidant capacity (A), super oxide dismutase (SOD) (B) and reduced glutathione (C) levels (n = 4). The cell pre-treated with CBD and then exposed with 6-OHDA for 24 h. **** P < 0.0001, *** P < 0.001, ** P < 0.01 and * P < 0.05 compared with control cells. $$$$ P < 0.0001, $$$ P < 0.001 and $ P < 0.05 compared with 6-OHDA-exposed cells. #, ##, and ### represent significant differences at probability levels of P < 0.05, P < 0.01, and P < 0.001, respectively, while ns signifies non-significant results.

Variance analysis of total antioxidant capacity [F (4, 15) = 37.83; P < 0.0001], SOD activity [F (4, 15) = 84.75; P < 0.0001], and GSH content [F (4, 15) = 77; P < 0.0001] indicated significant differences in different cell groups. Cellular antioxidant capacity, as the ability of cells to counteract oxidative stress, was significantly reduced by treatment of PC12 cells with 6-OHDA (19.25 ± 4.11 μM) compared to control cells (50.25 ± 5.90 μM) (P < 0.0001). Interestingly, PC12 cells pretreated with 5 and 10 μM CBD concentrations had significant increases in cellular antioxidant capacity (25.30 ± 4.03 μM and 39.75 ± 3.50 μM, respectively) compared to positive control cells (6-OHDA), indicating an improvement in antioxidant capacity by CBD. Also, 10 μM CBD improved cellular antioxidant capacity more than 5 μM of this compound (Figure 3A P = 0.039). The same trend was observed for SOD and GSH activities, and significant decreases were observed in SOD (Figure 3B) and GSH (Figure 3C) activities in cells exposed to 6-OHDA. However, pretreatment of PC12 cells with 5 and 10 μM CBD concentrations was able to prevent this decrease in SOD and GSH levels, indicating an improvement in cellular antioxidant enzymatic defense against 6-OHDA-induced oxidative stress. It is worth noting that 10 μM CBD significantly improved SOD (P = 0.009) and GSH (P = 0.010) levels compared to 5 μM CBD.

### 4.4. Gene Expression

**Figure 4.**
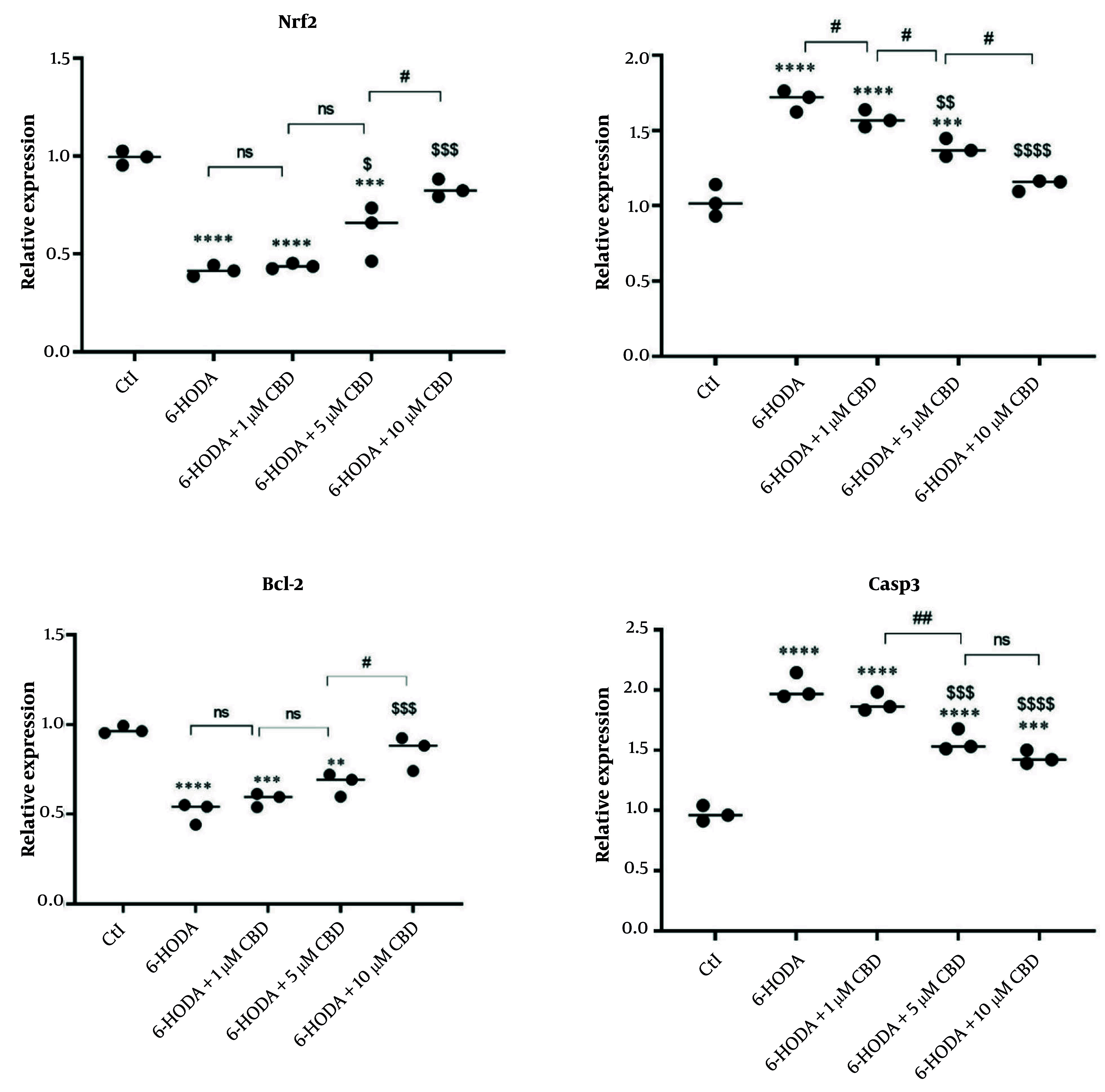
The effects of pretreatment of PC12 cell line with cannabidiol (CBD) on the *Nrf2*, *Bax*, *Bcl-2* and *Casp3* genes (n = 3). The cell pre-treated with CBD and then exposed with 6-OHDA. **** P < 0.0001, *** P < 0.001 and ** P < 0.01 compared with control cells. $$$$ P < 0.0001, $$$ P < 0.001, $$ P < 0.01 and $ P < 0.05 compared with 6-OHDA-exposed cells. #, and ## indicate significant differences at probability levels of P < 0.05, and P < 0.01, respectively, while ns denotes non-significant.

We evaluated the expression of the *Nrf2* gene, an important gene in the cellular antioxidant pathway, in PC12 cells, and the results showed that its expression was reduced in cells exposed to 6-OHDA, indicating a decrease in cellular antioxidant capacity. However, increased expression of this gene was observed in cells pretreated with 5 and 10 μM CBD and exposed to 6-OHDA, indicating that the neuroprotective properties of CBD are mediated through the *Nrf2* signaling pathway. Interestingly, pretreatment with 10 μM CBD resulted in the highest expression of this gene in cells exposed to 6-OHDA (Figure 4A). In addition, the expressions of *Bax* (Figure 4B), *Bcl-2* (Figure 4C), and *Casp3* (Figure 4D) genes, which are involved in the cell death signaling pathway (apoptosis), were measured, and the results indicated overexpressions of *Bax* and *Casp3* genes and a downregulation of *Bcl-2* in the 6-OHDA-exposed cells. However, CBD (5 and 10 μM) was able to significantly prevent the overexpression of *Bax* and *Casp3* and the downregulation of *Bcl-2*, indicating the high potential of CBD in preventing 6-OHDA-induced neurotoxicity.

## 5. Discussion

The findings of the present study indicated the neuroprotective effects of CBD against 6-OHDA neurotoxicity, as it prevented 6-OHDA-induced decreases in cell viability and increases in apoptosis. Also, CBD was able to prevent the increase in ROS and MDA contents in PC12 cells exposed to 6-OHDA by improving the total cellular antioxidant capacity and increasing the levels of SOD and GSH. Finally, the antioxidant effects of CBD were attributed to the overexpression of *Nrf2* and the anti-apoptotic effects by reducing the expressions of *Bax* and *Casp3* genes and upregulation of the *Bcl-2* gene. CBD, as one of the most important components of the hemp plant with various pharmacological effects, has attracted the attention of researchers. One of these pharmacological effects is the neuroprotective effects, and recently, it was found that 5 μM of this compound has protective effects against H2O2 neurotoxicity (33). Also, recently, the use of CBD as a supplement in a variety of neurodegenerative diseases has been mentioned (21, 34). Interestingly, CBD can prevent the negative effect of stress on hippocampal neurogenesis (35) and activation of microglia (36) that exacerbates inflammation. Also, its beneficial effects have been shown in mental diseases such as depression and schizophrenia (37). In the present study, the neuroprotective effects of CBD on 6-OHDA-induced neurotoxicity were also demonstrated, indicating that this compound can be recommended as an adjuvant treatment option in PD.

6-OHDA is a selective neurotoxin used to induce PD in cell and animal models (38). This compound increases ROS content through autoxidation of neurons, induces oxidative stress, and neurotoxicity (cell death) (39). In the present study, an increase in ROS content was also observed in cells exposed to 6-OHDA, which is similar to other findings in this field (39). In fact, the increase in ROS content is associated with the inhibition of Akt phosphorylation and phosphorylation of p38, which is associated with the activation of *Casp9* and *Casp3* (40). The activation of caspases is associated with the upregulation of *Bax* (pro-apoptotic) and the downregulation of *Bcl-2* (anti-apoptotic), all of which activate the cellular apoptosis pathway (41). In the present study, it was observed that exposure of cells to 6-OHDA was accompanied by overexpression of *Casp3* and *Bax* genes and decreased expression of *Bcl-2*, indicating activation of the apoptotic pathway in the cells. However, CBD significantly decreased the expression of pro-apoptotic *Bax* and *Casp3* genes in the cells and overexpressed the expression of anti-apoptotic *Bcl-2* gene, indicating that the neuroprotective impacts of CBD are through the reduction of the apoptosis signaling pathway. In addition, the reduction of apoptosis and improvement of viability of PC12 cells exposed to Aβ peptide and inhibition of *Casp3* activation by CBD have been reported in another study (42), which is similar to the findings of the present study. Therefore, CBD can be considered a potential neuroprotective compound.

Super oxide dismutase and GSH, as the main components of the antioxidant system, are considered the first barrier against oxidative stress-induced cellular damage (43). The results of the present study showed that CBD can prevent 6-OHDA-induced oxidative stress by improving the activities of GSH and SOD and increasing the total cellular antioxidant capacity. These results indicate the potential antioxidant effects of CBD, and it seems that the overexpression of *Nrf2* is one of the main mechanisms of the antioxidant effects of this compound. The *Nrf2* gene encodes a transcription factor that is involved in the expression of antioxidant proteins including SOD and glutathione (44), and its high expression is associated with protecting mitochondrial function and combating oxidative stress (45). Therefore, it appears that CBD improves cellular antioxidant capacity and SOD and GSH activities by upregulating the *Nrf2* gene.

However, other molecular pathways may be involved in the neuroprotective effects of CBD. For example, it has been shown that CBD can indirectly activate CB1R and increase the accumulation of endocannabinoids, thereby inducing neuroprotective effects (46). Also, by inhibiting microglial activation through CB2 receptor inhibition, it can reduce neuronal inflammation through downregulation of NF-κB (47). However, the neuroprotective effects of CBD are more likely mediated through the induction of improved antioxidant capacity and reduced inflammation, as demonstrated in the present study. It is worth noting that we used PC12 cells only, which are recommended by some authors for neurodegenerative diseases including PD (48), but they may not fully recapitulate the complexity of the in vivo condition. Thus, it seems that further studies, especially on different cell lines including SH-SY5Y and clinical studies, are needed in this regard. Furthermore, given that in this study CBD doses were selected below the cytotoxic concentrations, the effects of CBD on control PC12 cells were not evaluated. Thus, it is recommended that future studies also evaluate the effects of different doses of CBD in control cell and animal models. However, this study, for the first time, showed that CBD can exert neuroprotective effects via upregulation of *Nrf2* pathway's genes, which is the main novelty of the current study. The findings of this research could be useful in better understanding the mechanisms of action of CBD and consequently in developing new therapeutic approaches in PD.

### 5.1. Conclusions

In general, it was concluded that CBD has neuroprotective impacts against 6-OHDA-induced neurotoxicity due to its antioxidant properties that mediate via the *Nrf2* signaling pathway. However, more studies for evaluating the CBD pharmacokinetics, drug interactions, and potential side effects are needed in this regard.

## Data Availability

The dataset presented in the study is available upon request from the corresponding author during submission or after publication. Due to the format of the statistical analysis software and the volume of data, the data are not publicly available.
